# Virtual Reality and Higher Education Sporting Events: Social Anxiety Perception as an Outcome of VR Simulation

**DOI:** 10.3390/bs14080695

**Published:** 2024-08-10

**Authors:** Kyu-Soo Chung, Chad Goebert, John David Johnson

**Affiliations:** Department of Exercise Science and Sport Management, Kennesaw State University, Kennesaw, GA 30144, USA; kchung2@kennesaw.edu (K.-S.C.); jjohn235@kennesaw.edu (J.D.J.)

**Keywords:** immersive technology, exposure therapy, innovation, consumer behavior

## Abstract

Background: This study investigates the relationship between Virtual Reality Exposure Therapy (VRET) and social anxiety in sport environments. Social anxiety is a mental health condition that manifests people’s intense fear of being watched and judged by others and worrying about humiliation It is important to research potential tools like VRET that could help to mitigate the impact of social anxiety as people with social anxiety often avoid attending live events due to the venue’s sensory stimuli and the social encounters they anticipate. VR simulation could allow socially anxious individuals to fully experience a sporting event simulation minus the anxiety induced by potential social encounters. VR’s therapeutic effects on social anxiety should be explored when considering several findings of VR intervention to mental health. Aim: The study aims to assess the impact of exposing socially anxious people to a virtual sporting game by measuring their levels of social anxiety, team identification, and intentions to attend a live sporting event before and after the VR exposure. Due to VR’s positive experience, social anxiety is expected to decrease. However, team identification and intentions to attend live sporting events are expected to increase because of VR’s ability to develop sport fanship. Method: Fourteen students with symptoms of social anxiety participated in the study. To create the VR simulation stimuli, the researchers used six 360° cameras to record an NCAA Division-I women’s volleyball game. Participants experienced the sporting event via VR simulation. Data were analyzed via one-group pre- and post-comparison. Results and Conclusions: Significant results were found for behavioral intentions of participants after experiencing the simulation. Social anxiety’s difference was negative 0.22, t(13) = 3.47, *p* < 0.01. After watching the game in VR, the respondents’ social anxiety decreased significantly. Team identification’s difference was 0.53, t(13) = −3.56, *p* < 0.01. Lastly, event visit intentions’ difference was 0.24, t(13) = −2.35, *p* < 0.05. Team identification and intentions to visit a sporting event rose significantly after viewing the game in VR.

## 1. Background

Live sporting events are designed to be full of entertainment and social interactions which stimulate event attendees with various sensory stimuli [1]. Many people seek out such an atmosphere, but not every group of people [2]. For example, socially anxious individuals would feel uncomfortable encountering the various social interactions that occur at a live sporting event [3]. Therefore, their visits to live sporting events are limited. Recent studies have indicated that social anxiety is on the rise and is impacting more and more individuals each year [4]. With that in mind, it is important to understand how Virtual Reality (VR) can help socially anxious individuals alleviate their anxiety and develop their sports fanship. This is a novel area that has yet to be explored; thus, the findings suggest an inclusive way to use VR for reaching more diverse populations.

Virtual reality (VR) can be a way for socially anxious people to experience a live sporting event by staying in a digitally simulated environment as if they were at the real venue. VR’s experiential aspect could satisfy the sport needs of socially anxious people and alleviate their anxiety. However, at this time, we have little knowledge regarding their sport VR experiences. Therefore, the current study examines the effect of watching a sports game via VR simulation on sports fanship for socially anxious people and examines the effect of watching a sports game via VR simulation on anxiety levels for socially anxious people.

Social Anxiety Disorder (SAD) is a mental health condition that manifests people’s intense fear of being watched and judged by others and worrying about humiliation [5]. Also referred to as social phobia, SAD can affect individuals’ daily activities, job functions, and even leisure and recreational activities. There have been substantial systematic approaches to scale different degrees of SAD to more precisely understand the SAD’s diverse symptoms [5]. For example, Connor and colleagues (2000) developed the Social Phobia Inventory (SPIN), a self-rating survey consisting of 17 items that measure the spectrum of fear, avoidance, and physiological components of social phobia. The SPIN inventory was tested on 353 subjects, consisting of two control and three social phobia clinical groups, to establish the scale’s psychometric validation. The scale demonstrated favorable results regarding test–retest reliability, internal consistency, and convergent and divergent validity [6].

This indicates that SPIN plays several roles in distinguishing people with a diagnosis of social phobia from those without it, in indicating the degree of social phobia symptoms, in detecting changes in symptoms over time, in identifying the threshold point of social phobia, and in facilitating the discovery of the effects of different interventions and treatments on social phobia [6]. Socially anxious people are likely to avoid attending a live sporting event due to the social encounters they anticipate [3]. The current study adopts SPIN to measure such a tendency. 

One of the most impactful therapies for individuals who struggle with social anxiety is exposure therapy [7]. Role-playing games have also been used successfully as a form of exposure therapy for mental health conditions [8]. A more recent form of exposure therapy that involves role-playing is Virtual Reality Exposure Therapy (VRET). VR provides a sense of presence comparable to a real-world setting and can elicit authentic emotional responses from users [9]. VR has been shown to provide realistic simulations that elicit real-world emotional reactions for individuals with social anxiety [10,11]. Thanks to this realism, VRET is used to treat those with phobias or social anxiety by exposing them to social situations they fear in VR [12].

Thanks to technological advances, VRET controls the simulated content and duration according to the symptoms of social anxiety [13]. The current study adopts VRET in which socially anxious people virtually explore a sporting game in 360°, observing how attendees cheer for teams and athletes and socially interact. Controlled environments that afford anonymity, such as our VR simulation, have been shown to reduce anxiety [14]. In a sporting context, VR’s therapeutic effect would be a reduction, after watching the VR, of social anxiousness. 

VR is a platform by which socially anxious individuals can fully enjoy a simulated sporting game minus the anxiety induced by potential social encounters. VR can provide a close-to-reality simulation to allow users to participate via role-play in an experience or environment that provokes feeling or emotions and thus prepare them for that real-world situation [15]. Moreover, researchers have reported VR’s positive therapeutic effects on mental health [16,17]. It is worth investigating sport VR’s therapeutic function for social anxiety. Thus, the study measures socially anxious people’s anxiety levels pre- and post-viewing of a sporting event in VR, generating the first hypothesis.

**H-1.**  *Would socially anxious people’s anxiety levels be decreased after watching a sporting game in VR?*

Sport fanship is a social, psychological, and cultural concept that theorizes how sport fans support and follow their favorite teams and athletes [18]. As for the psychological aspect, team identification conceptualizes sport fans’ psychological connection or bond between sport fans and the team and athletes they identify with [18]. Team identification is the central experience of sports fans, playing a crucial role in explaining how much sport fans commit themselves to the team and its athletes [19]. Sport fanship’s behavioral aspect primarily concerns sport fans’ consumption, exemplified by game visits, media usage, or merchandise acquisition [20]. Another behavioral aspect is consumption intentions, such as sport fans’ intentions to visit a sporting game [21].

Studies [22,23,24] have found that VR helps sport fans develop various characteristics in their fanship. For example, Kim and Ko [25] found that VR enhanced the consumption of sport media and the loyalty to the organization or team displayed in the media. Similarly, Uhm et al. [26] found that the VR’s sense of presence positively impacted the users’ attitude towards the sport. Given the positive impact of VR on sport fanship, the current study examines how socially anxious people’s team identification and intentions to visit a sporting game would change pre- and post-viewing of a sporting event in VR. Finding the increases of team identification and sporting game visit intentions would reveal the VR’s utilization for socially anxious people’s sport fanship development. The study puts forward the second and third hypotheses.

**H-2/3.**  *Would socially anxious people’s team identification and intentions to visit a sporting game be increased after watching a sporting game in VR?*

The study aims to assess the impact of exposing socially anxious people to a virtual sporting game by measuring their levels of social anxiety, team identification, and intentions to attend a live sporting event before and after the VR exposure. By comparing the scores obtained before and after the exposure, the study seeks to test the hypotheses concerning changes in social anxiety, team identification, and visit intentions as a consequence of the VR experience. 

## 2. Method

### 2.1. Participants 

Housed in the researchers’ institution is a special-needs academic program. The program indicates its purpose as “a fully inclusive post-secondary college education and experience to students with different intellectual or developmental abilities”. The study’s cohort comprises individuals possessing diverse intellectual or developmental capabilities, all of whom are members of a program specifically structured to promote social integration and career exploration. To qualify for this program, students must meet the eligibility requirements for special-needs education. Recruiting students from the special-needs program provides contextual validity for target sampling, allowing the study to measure anxiety levels within this specific population pre- and post-VR exposure.

With the study’s IRB approval and the program’s permission, the researcher—a program instructor who was not a researcher in the study—recruited 14 students who showed some symptoms of social anxiety. The instructor had daily interactions with the program’s students; therefore, the instructor was aware of the students’ social behaviors. Further, the researcher contacted the students’ legal guardians for their permission. All subjects and their guardians gave their informed consent for inclusion before they participated in the study. The study was conducted and the protocol was approved by the IRB the researcher’s institution (Study number 18-362).

Of 14 students, nine were male (64.3%), and five were female (35.7%). Their average age was 22.1 years old, ranging from 19 to 27. In the initial assessment, on a scale of 5 points, their social anxiety level was 1.69 (*SD* = 0.5), which described their anxiety degree on the anxiety spectrum. This score serves as their position on the anxiety spectrum, though it does not specify the reasons behind their anxiety symptoms. The demonstrated level of anxiety reinforces our sampling approach to the students enrolled in the special-needs academic program.

### 2.2. Data Collection

The meetings between the researcher and the participants were scheduled individually, with only one participant present at a time. The meetings were held in the classroom of the special-needs program, which provided a comfortable and familiar environment for the participants. To alleviate any psychological discomfort related to anticipating the VR experience, the researcher spent some time engaging in ice-breaking activities with each participant. These activities included small talk, such as discussing the weather and inquiring about their daily activities and interests. This process ensured that external influence was minimized and the environment was well-controlled. 

For the pre-examination, the researcher read each question and marked the participants’ answers on the survey. When the participants had difficulty understanding the question, the researcher explained the question further. The researcher then assisted the participants in wearing the VR headset and watching the game in VR. During the 5-min simulation, the participants were entertained by watching the game and had the opportunity to explore the game’s 360° atmosphere consisting of virtual spectators around them and the entire venue without screen boundary. However, it is not clear that participants fully explored the entirety of the 360° game atmosphere available in the simulation. After watching, the participant underwent a post-examination to answer the same questions for any change. This process typically took an hour per participant.

### 2.3. VR Stimuli and Technology Specification

To create the VR stimuli, the researchers used six 360° cameras to record an NCAA Division-I women’s volleyball game. The audio was recorded on stereo devices. The six VR compatible video files were edited with Kolor’s Autopano Giga 4.4 software and combined into a three-minute segment of the game. The video is available at https://www.youtube.com/watch?v=VdxNfW3xxgo&feature=youtu.be (accessed on 7 August 2024). Table 1 includes more details on device specifications.

### 2.4. Measures

The original version of the SPIN questionnaire comprises seventeen items designed to evaluate social anxiety stemming from active participation in social interactions, particularly those involving authority figures or experiencing criticism. These items gauge individuals’ anxiety levels concerning their fear, social avoidance behaviors, and physiological reactions. These symptoms are particularly relevant in settings, such as classrooms or offices, where more formal interactions occur.

Out of the original 17 items, this study selectively incorporated seven items to assess respondents’ social anxiety concerning hypothetical social situations they might encounter during a sporting event. For example, one item, “Talking to strangers in a sporting event would scare me”, gauged their fear levels. Another item, “In a sporting event, I would avoid interacting with people I don’t know”, assessed their behavioral inclination to avoid social interactions. A measure of their physiological reactions included, “Heart palpitations would bother me when I am around people in a sporting event”. In summary, four of these adopted items were designed to measure fear, two evaluated behavioral tendencies related to social avoidance, and one assessed physiological reactions. This item selection retained the three core categories of social anxiety—emotional fear, social avoidance behavior, and physiological reaction—present in the original SPIN version. Table 2 provides detailed statements related to these adopted items.

The deliberate choice to omit the remaining ten items from the original SPIN version stemmed from their lack of relevance to the sporting event context under examination. It is important to note that this decision aligns with several previous research to select specific subsets of the SPIN based on the unique requirements of their particular research contexts [27,28]. Moreover, it is worth highlighting that Campbell-Sills et al. [29] conducted research demonstrating that the SPIN could be effectively condensed without compromising its validity, further supporting the rationale for our selective use of items.

To assess the compatibility between the original SPIN and the adopted items regarding three sub-sections, the researchers factored the seven selected items using exploratory factor analysis (EFA) with varimax rotation. Due to only 14 samples in the study, the study ensured the suitability of the data for EFA by assessing data normality, which yielded skewness of 0.78 and kurtosis of 0.98. Additionally, EFA revealed the presence of three factors: anxious feelings (26.65%), avoidance behaviors (21.62%), and physiological responses (20.29%), collectively accounting for 68.56% of the total variance. A robust structural alignment between the selected items and the original SPIN was indicated. 

For team identification, the current study used three items to measure the respondents’ team identification. Three items were also used to measure the respondents’ intentions to attend a sporting event. Each item was asked on a 5-point Likert-type scale, ranging from 1 (not at all) through 3 (somewhat) to 5 (extremely). All items’ exact wording is shown in Table 2. 

Due to the study’s small sample size (N = 14), each construct’s correlation between pre- and post-scores is provided for items’ reliability [30]. The correlation is 0.87 in social anxiety (*p* < 0.001). The correlation is 0.90 in team identification (*p* < 0.001). Lastly, the correlation is 0.88 in event visit intention (*p* < 0.001). In addition, each construct’s Principal Component Factor Analysis results in 69% variances explained for social anxiety, ranging from 0.61 to 0.80 in item communality. Seventy-one percent of variances are explained for team identification, ranging from 0.54 to 0.81. The variances for event visit intention are 87%, ranging from 0.80 to 0.90. All these statistics suggest items’ reliability and validity.

### 2.5. Data Analysis

Collected data were coded into a statistical program, SPSS 28. Pre- and post-scores were compared on social anxiety, team identification, and intentions to visit a sporting event in a paired *T*-test. The test secures the differences between the two scores and their two-sided statistical significance at a 0.05 level. The test also secures Cohen’s d indicator to suggest the difference’s power. 

## 3. Results

Social anxiety’s score change before (1.69, *SD* = 0.50) and after (1.47, *SD* = 0.45) VR exposure was negative 0.22, *t*(13) = 3.47, *p* = 0.004. The difference’s effect (i.e., Cohen’s d) was 0.24, which is considered small. After watching the game in VR, the respondents’ social anxiety decreased significantly (H-1 accepted). Team identification’s score change before (2.33, *SD* = 1.19) and after (2.86, *SD* = 1.21) VR exposure was 0.53, *t*(13) = −3.56, *p* = 0.003. The effect was 0.55, which is a medium impact. Lastly, the score change in event visit intentions was 0.24, *t*(13) = −2.35, *p =* 0.035. The effect was small, 0.38. The respondents’ team identification and intentions to visit a sporting event before (3.38, *SD* = 0.79) and after (3.62, *SD* = 0.78) VR exposure rose significantly after viewing the game in VR (H-2/3 accepted). Each construct’s pre- and post-scores and corresponding standard deviations are shown in Table 1.

## 4. Discussion

Foa and Kozak’s [31] emotional processing theory is a conceptual foundation of exposure therapy for social anxiety. The theory explains that people perceive an imposed situation’s stimuli, interpret the meaning of the stimuli to elicit their emotions, and behave to escape or seek the stimuli according to the emotions. The current study’s exposure immersed participants for 5 min in a sporting environment displayed in VR. Participants experienced the stimuli and processed them, resulting in psychological and behavioral outcomes accordingly. Given this, the current study’s Virtual Reality Exposure Therapy (VRET) was sufficient to apply to socially anxious people. 

One of the study’s objectives is to find the therapeutic benefit of VRET, which is customized to the sport spectating context. VRET simulates real-life situations for socially anxious people [13]. To accrue more therapeutic benefits, VRET controls the simulated content and duration to deliver the sense of presence (i.e., reality) in the virtual environment. The current study’s VR stimuli were not manipulated according to the participants’ specific social anxiety symptoms; instead, the participants were able to gain a sense of the venue’s atmosphere, and autonomously had the opportunity to explore every corner of the sporting venue. Experiencing the venue in VR seemed to familiarize the study participants with the event’s atmosphere, as evidenced by their moderately reduced anxiety (H-1 accepted). The study’s use of a sporting game was effective for socially anxious people by enabling them to virtually experience the game venue and its cheering atmosphere. 

While the effect of VR was significant on anxiety levels, it was small in magnitude. The researchers speculate that this could be due to the respondents’ tendency to underreport their initial anxiety levels, possibly due to social desirability [32]. The respondents might have been reluctant to report their anxiety levels accurately before VR exposure, leading to a smaller effect size than the actual difference in anxiety levels. Additionally, the current study’s VRET did not involve any specific behavioral intervention, which could also be a reason for the small effect size. Nevertheless, the results indicate that VR exposure significantly reduced respondents’ anxiety levels. This suggests that even without specific interventions, a virtual experience of a sporting event’s environment could help prepare socially anxious individuals by aligning their expectations with the actual sporting environment. 

It can be argued that the respondents’ low initial anxiety levels (indicated by a mean score of 1.69, *SD* = 0.50 on a five-point Likert-type scale in Table 2) may not fully capture the social anxiety symptoms. However, the researchers argue that even a low score reflects the respondents’ social anxiety because social anxiety symptoms should be on the spectrum. To support this argument, the researchers surveyed 24 students from the same institution but from the academy about their social anxiety. This group consisted of 19 males and 5 females, with an average age of 21.7. They were asked the same survey questions on a five-point Likert-type scale. The average score for this group was 1.08 (*SD* = 0.41), which is lower than the mean score of 1.69. To further check how this group’s social anxiety would be statistically lower than that of the study’s respondents, the researchers conducted an independent T-test. The results showed a significant difference, t(36) = −4.10, *p* < 0.001, indicating that the respondents’ initial social anxiety score was statistically higher than that of the group with no social anxiety symptoms. That is, since social anxiety should be viewed on the spectrum, a certain magnitude does not point to the absolute status of social anxiety. The study’s VRET effectively reduced social anxiety levels but moderately. 

The study also aimed to test if the study’s VRET would help socially anxious people develop their team identification and intentions to visit a sporting event. The study finds that socially anxious people increased their team identification (H-2 accepted) and intentions to visit a sporting event after watching a sporting game in VR (H-3 accepted). Sport studies have found that a sport fan’s fanship and sociability are substantially related to one another [21,33]. For example, sport fans often present their fanship by wearing a favorite team’s jersey or talking about their teams and associated athletes. This behavior often gives rise to interactions in which socially anxious people talk about their teams and athletes. That is, socially anxious people’s sport fanship might not be a direct trigger to cause social interactions but could work as motivation to connect with others, especially when they have favorite teams and athletes.

The effect of VRET was medium on the difference in team identification. Moreover, the study’s VRET had a minor impact on the difference in event visit intentions. Similar to the interpretation of VRET’s effect on social anxiety, the researchers consider the current study’s lack of behavioral intervention in VRET for small and medium size effects. Socially anxious individuals’ team identification is a psychological construct that they can shape easily by merely experiencing the game’s excitement and entertained atmosphere in VR, causing the VR’s effect to be greater than small. However, forming behavioral intentions to visit the event is influenced by various factors such as resources and barriers. Therefore, the VR’s effect was significant but small on the intentions. 

The study found the VRET’s function to develop team identification and sporting event visit intention among socially anxious people. This finding opens the discussions of socially anxious people’s sport fanship development. Their anxiousness often prevents them from engaging in social interactions and enjoying leisure activities, depriving them of opportunities to further their commitment to the activities. This deprivation can be exemplified by having little or no experience attending live sporting events. The VR’s sense of presence plays a role in filling the void of a live sporting experience. More systematic approaches should find how VR can enhance socially anxious people’s entertainment and leisure consumption. Moreover, it is worth investigating how socially anxious people’s sport fanship helps them ease their anxiety and how VR facilitates the process. 

### Future Studies and Limitations

This investigation prioritized the examination of shared symptoms of social anxiety among the participants, rather than the individualized causes underlying their anxiety. This focus was strategic, aiming to assess the efficacy of VR technologies in mitigating social anxiety within highly social environments such as sporting events, which are characterized by explicit social stimuli.

While individual contributory factors of each participant’s anxiety were not the focus of this study, acknowledging these factors remains crucial. The present study emphasizes the commonality of symptoms and their management within a specified context, establishing a foundation for future research. Future studies may seek to integrate the management of social anxiety symptoms with a deeper understanding of their causative factors.

Previous studies have pointed out that VRET’s physiological reactions are not quite as intense as in non-simulated and real-life exposure (i.e., vivo exposure) reactions [34]. The current study used one item to ask participants about physiological reactions to feeling anxiety; however, their answers might not precisely reflect their actual physiological reactions. Future studies should consider the direct measure of physiological reactions in applying VRET simulations, especially when being exposed to a sporting event. A future study could also incorporate the use of a control in the form of a video with similar content to the VR simulations. This could demonstrate whether or not there was a greater easing of anxiety by the VR simulation than the simple video. Further, future studies could track where participants moved throughout the virtual simulation or could ask participants what areas of the simulation they explored. This would give the researchers a greater understanding of the consistency and thoroughness of the participant utilization of the virtual atmosphere. A live app could also be used rather than a video.

Another limitation of the current study is also the relatively small sample size (14) and the difference in representation of genders in the study (9 male, 5 female). Future studies could attempt to adjust for this by recruiting a larger and more heterogenous sample. 

Although the current study did not include a comparison between a socially anxious group and a group without anxiety regarding fanship development, the researchers hypothesize that the baseline group would also develop their sport fanship if they watch a sporting game in VR. It is, therefore, a limitation of this study that control groups were not utilized. Future studies into this topic would be made stronger by the inclusion of control groups. Additionally, it is essential to note that the VR exposure duration in the study was only three minutes. Thus, future research should investigate the duration of sport VR exposure and compare it to a baseline group to confirm the impact of VR on the development of fanship in socially anxious individuals. Moreover, a future study could explore how the effect of sport VRET intersects with prior spectating experience by comparing a socially anxious group that has attended a live sporting event with a group that has not had that experience.

Many VRET simulations use avatars to impose real-life situations on socially anxious people; it is possible that therapy participants would feel less pressure and fear from the virtual figures than those with actual persons. While the current study’s VR does not include virtual figures, it is worth exploring how sport-specific avatars in VRET would affect a person’s level of social anxiety. For example, manipulating the VR figure’s facial expressions, characters, voice quality, gender, and kinetic movement can affect socially anxious people’s clinical outcomes. Knowing this would provide meaningful practical implications regarding how a sporting venue could be made more approachable for people with social anxiety, especially when they have to interact with staff at the box office or concessions. Further, teachers and specialists could incorporate VRET into their programs to facilitate the gradual acclimation of socially anxious individuals to social environments, such as school events and activities. By using VR simulations of such environments, educators can provide a safe and controlled setting for students to experience their surroundings, ultimately reducing their anxiety and improving their overall involvement in their program.

Finally, while VRET’s effectiveness has been addressed in various social contexts [34], such as public speaking, its effectiveness at dealing with the anxiety caused in sport and leisure contexts has yet to be explored. People with social anxiety could, for example, become fearful when they have to interact with others while ordering food or finding restrooms and seats. They may wish to avoid encountering other spectators next to them. This tendency would become stronger when a sporting venue became loud with excitement. Future studies should customize VRET according to specific sport contexts so that more situation-oriented approaches and context-specific characteristics are possible in VRET’s simulations. 

## 5. Conclusions

This study was the first of its kind to investigate the effect of watching a sports game via VR simulation on sport fanship for socially anxious people and the effect of watching a sport game via VR simulation on anxiety levels for socially anxious people in a sport or leisure environment. The finding that participants indicated a lower anxiety of a sport atmosphere after their experience with VRET is a promising result when considering the challenge of how to help individuals with social anxiety to acclimate to those environments. This finding could lead to opportunities that would allow socially anxious individuals to experience a sporting venue through VRET simulation and become familiar with the sights and sounds of that type of venue before attending in person. 

The second and third findings that participants increased their team identification and intentions to visit a sporting event after their experience with that team and venue in VR are equally encouraging. Attendance at a sporting event is a key driver of cultivating strong and enduring team identification and sport fandom [35]. If a portion of the market, socially anxious individuals, that did not attend sporting events previously due to their anxiety, were able to attend sporting events in person after having experienced a similar event in a VR simulation, it would allow for them to potentially reach levels of fandom and attachment to a team that they previously did not have. This finding is a positive not only for the socially anxious individual that can now form that connection to a team, but also to the team or organization that now would reach an audience in a way that they previously could not reach before. 

As the reported number of individuals with social anxiety continues to rise [4], the results of this study hold promise for the use of VR simulation in assisting socially anxious individuals in their attempts to attend events in a sport or leisure setting. VR simulation may well be a gateway to allow socially anxious individuals to strengthen their team identification and serve as a motivation to allow those individuals to connect and unite with others in their shared fandom of a favorite team or athlete and attend events.

## Figures and Tables

**Table 1:** Device technology specifications.

	Device	Specification
VR Headset	Samsung Gear VR Oculus	Android 1280 by 1440 per eye resolution
Smartphone	Samsung Galaxy S6 Edge	5.1 inches screen size 2560 by 1440 pixels resolution per inch
Headphone	JBL E45BT Wireless	20 Hz–20 kHz frequency range

**Table 2:** Items’ descriptions and scores.

Construct	Items on a Five-Point Likert-Type Scale [From 1 (Not at All) through 3 (Somewhat) to 5 (Extremely)]
Social Anxiety	Attending a sporting event would scare me.
	In a sporting event, I would avoid interacting with people I don’t know.
	I would avoid going to mass-gathering events like sport games.
	Heart palpitations would bother me when I am around people in a sporting event.
	Talking to strangers in a sporting event would scare me.
	I am afraid of doing things when people might be watching.
	Being embarrassed or looking stupid are among my worst fears.
Team Identification	How important is it that the team wins?
	How important is it to you to be a fan of the team?
	How strongly do you feel good about being a fan of the team?
Visit Intention	I intend to attend a collegiate sporting event.
	I intend to attend the university’s sporting event.
	I intend to attend the university’s volleyball match.

## Data Availability

The datasets presented in this article are not readily available due to the nature of the population that participated in the study. Requests to access the datasets should be directed to kchung2@kennesaw.edu.
